# Ligand-dependent corepressor suppresses angiogenesis and macrophage infiltration in gliobl astoma microenvironment via MCP-1 down-regulation

**DOI:** 10.1186/s12896-026-01166-6

**Published:** 2026-05-18

**Authors:** Shu-Kai Lin, Tai-Qin Li, Xiang-Lan Luo, Yi-Chi Zhang, Jun-Xue Qian, Shi-Lu Huang, Ming Yang, Ke-Gang Linghu, Jun Dong

**Affiliations:** 1 Department of Neurosurgery, The Second Affiliated Hospital of Soochow University, Suzhou, Jiangsu China; 2 Department of Neurosurgery, Sanya Central Hospital (The Third People’s Hospital of Hainan Province), Sanya, Hainan China; 3 The High Efficacy Application of Natural Medicinal Resources Engineering Center of Guizhou Province, State Key Laboratory of Discovery and Utilization of Functional Components in Traditional Chinese Medicine & School of Pharmaceutical Sciences, Guizhou Medical University, Guiyang, Guizhou China; 4 Department of Pathology and Intelligent Pathology Institute, The First Affiliated Hospital of USTC, Division of Life Sciences and Medicine, University of Science and Technology of China, Hefei, Anhui China; 5 Institute of Pathology and Southwest Cancer Center, Southwest Hospital, Third Military Medical University (Army Medical University), Key Laboratory of Tumor Immunopathology, Ministry of Education of China, Chongqing, China; 6 Department of Neurosurgery, The Affiliated Hospital of Guizhou Medical University, Guiyang, Guizhou China

**Keywords:** Ligand-dependent nuclear receptor corepressor, Glioblastoma, Chemokines, Tumor-associated macrophages, Angiogenesis, Tumor microenvironment

## Abstract

**Background:**

Glioblastoma is one of the most aggressive and lethal forms of brain cancer, characterized by rapid angiogenesis and infiltration of tumor-associated macrophages in the tumor microenvironment (TME). Given the limited efficacy of single-targeting tumor cell therapies against glioblastoma, regulating the TME has emerged as a promising strategy to improve therapeutic outcomes.

**Materials and methods:**

The Cancer Genome Atlas (TCGA) was used to assess the association between ligand-dependent nuclear receptor corepressor (LCoR) expression and glioblastoma. LCoR expression in patient glioma specimens was examined by qRT‑PCR and Western blot. LCoR was knocked down or overexpressed in glioblastoma cell lines (SF295, SNB19) using lentiviral vectors, followed by functional assays for proliferation, migration, invasion, angiogenesis, chemokine secretion, and monocyte migration. Subcutaneous and orthotopic mouse models were established to evaluate tumor growth, angiogenesis, and macrophage infiltration via immunohistochemistry and immunofluorescence. Transcriptome sequencing was performed on LCoR‑overexpressing cells to explore underlying mechanisms. In addition, mice were treated with the small-molecule compound deoxynyboquinone to assess its therapeutic effect via LCoR‑mediated regulation.

**Results:**

TCGA analysis reveals lower LCoR expression in glioblastoma than in normal tissues, correlating with improved patient survival. Clinical samples further confirm reduced LCoR expression in tumor versus peri-tumor tissues. LCoR knockdown significantly enhanced the proliferation, migration, and invasion of glioblastoma cells, whereas LCoR overexpression inhibited these malignant phenotypes. Mechanistically, LCoR suppressed VEGF and HIF-1α expression in tumor cells, thereby reducing angiogenesis and tumor sphere formation. Transcriptome sequencing revealed that LCoR is involved in immune regulation. Further experiments demonstrated that LCoR inhibited chemokine secretion, thereby reducing monocyte migration and macrophage M2 polarization. In subcutaneous and orthotopic glioblastoma mouse models, LCoR overexpression suppressed tumor growth by inhibiting angiogenesis and macrophage M2 polarization. Treatment with the deoxynyboquinone reduced angiogenesis and macrophage infiltration in the TME through LCoR-mediated downregulation of MCP-1, thereby blocking glioblastoma progression.

**Conclusion:**

LCoR serves as a potential therapeutic target against glioblastoma. Upregulating LCoR can inhibit glioblastoma development by suppressing angiogenesis and immune evasion within the TME.

**Supplementary Information:**

The online version contains supplementary material available at 10.1186/s12896-026-01166-6.

## Graphical Abstract

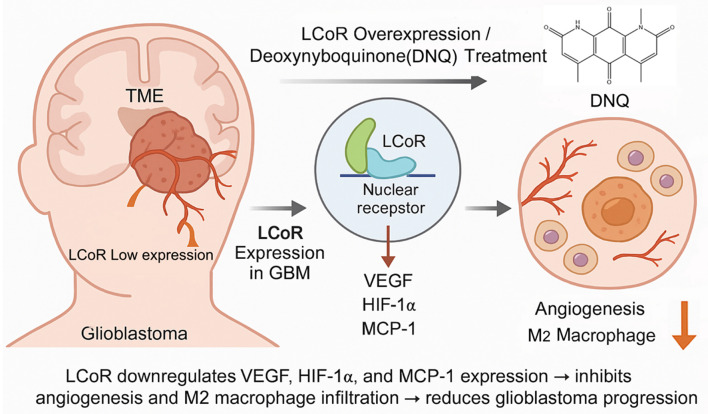

## Introduction

Glioblastoma is the most common and aggressive primary malignant brain tumor, characterized by rapid growth, extensive infiltration, and resistance to standard therapies such as surgery, radiotherapy, and chemotherapy [1]. Despite advancements in treatment modalities, the prognosis for glioblastoma patients remains dismal, with a median survival of approximately 14–16 months. Aggressive behaviors of glioblastoma are closely related to active mutual interactions of tumor cells with tumor microenvironment (TME), a dynamic network of immune cells, stromal cells, and signaling molecules that collectively support tumor progression and immune evasion [2].

Within the TME, tumor-associated macrophages (TAMs) play a pivotal role in shaping the immunosuppressive landscape of glioblastoma [3]. TAMs are commonly classified as pro-inflammatory M1 macrophages and anti-inflammatory M2 macrophages. M1 macrophages are characterized by their production of pro-inflammatory cytokines (e.g., TNF-α, IL-6), their capacity to present antigens, and their ability to mount an anti-tumor immune response. In contrast, M2 macrophages are associated with wound healing, tissue remodeling, and immune suppression, which contribute to tumor progression. In glioblastoma, TAMs are predominantly skewed toward the M2 phenotype, contributing to tumor growth, invasion, and immune evasion [4]. Strategies to reprogram TAMs from an M2 to an M1 phenotype, thereby restoring their anti-tumor activity, have gained traction as a potential therapeutic approach [5].

Additionally, hypoxic conditions within the tumor microenvironment drive the upregulation of pro-angiogenic factors, such as vascular endothelial growth factor (VEGF), hypoxia-inducible factor-1α (HIF-1α), and angiopoietins, which activate endothelial cells and promote abnormal vessel formation [6]. These newly formed vessels are structurally disorganized, leaky, and inefficient, further exacerbating hypoxia and creating a vicious cycle that perpetuates tumor aggressiveness. Aberrant angiogenesis in glioblastoma also facilitates immune evasion and treatment resistance [7]. The chaotic vasculature limits immune cell infiltration while supporting immunosuppressive cell populations, such as TAM, which secrete additional pro-angiogenic cytokines. Notably, crosstalk between endothelial cells and glioblastoma stem cells enhances tumor cell survival and self-renewal, contributing to recurrence [8]. Targeting angiogenesis in combination with immunotherapies or microenvironment-modulating agents represents a promising strategy to disrupt this multifaceted interplay and improve outcomes in this devastating disease.

Ligand-dependent nuclear receptor corepressor (LCoR) is a class of transcriptional regulators that modulate gene expression by repressing the activity of transcription factors [9]. It was first identified as a cofactor that suppresses the activity of estrogen receptor α (ERα) by recruiting histone deacetylases (HDACs) and other chromatin-modifying enzymes [10]. In cancer, the dysregulation of transcriptional regulators like LCoR can profoundly influence tumor progression and metastasis. For example, LCoR has been implicated in hormone-sensitive cancers such as breast and prostate cancer, where it modulates the activity of nuclear receptors [11, 12]. However, its role in glioblastoma, a malignancy not typically associated with hormone dependency, has not been fully demonstrated.

In this study, our results reveal that LCoR exerts tumor-suppressive effects in glioblastoma by interfering with angiogenesis and immune evasion. The discovery of LCoR as a key regulator of angiogenesis and TAM polarization in the tumor microenvironment represents a significant advancement in our understanding of glioblastoma biology. By simultaneously targeting tumor cell proliferation and the immunosuppressive TME, LCoR holds promise as a novel therapeutic target. Additionally, our studies suggest that deoxynyboquinone (DNQ), an LCoR upregulator, suppresses angiogenesis and macrophage infiltration in TME by downregulating chemokines. The identification of DNQ as a small molecule that upregulates LCoR expression underscores the translational potential of targeting this pathway.

## Materials and methods

### Chemicals and reagents

Deoxynyboquinone (DNQ) was dissolved in dimethyl sulfoxide (DMSO) to prepare a 10 mM stock solution, which was then aliquoted and stored at -80 °C to avoid repeated freeze-thaw cycles. Phorbol 12-myristate 13-acetate (PMA) was dissolved in DMSO at a stock concentration of 1 mg/mL and stored at -20 °C protected from light. All other reagents were stored at room temperature or 4 °C as recommended by the manufacturers, and all operations involving cell experiments were performed under sterile conditions. Working solutions of all reagents were freshly prepared prior to each experiment to ensure the stability and consistency of experimental results. The chemicals and biological reagents used in this study are listed in Table 1.

**Table 1:** The chemicals and biological reagents used in this study

Name	Source	Identifier
Deoxynyboquinone (DNQ)	MCE (Shanghai, China)	#HY-108,992
Cell Counting Kit-8 solution	Beyotime (Shanghai, China)	#C0038
Phorbol 12-myristate 13-acetate (PMA)	Sigma (St. Louis, MO, USA)	#P8139
DMSO	Sigma (St. Louis, MO, USA)	#D1435
BCA protein assay kit	Solarbio (Beijing, China)	#PC0020
Fetal bovine serum (FBS)	Cellmax (Shanghai, China)	#SA301.02.V
2’,7’-bis-(2-carboxyethyl)-5-(and-6)-Carboxyfluorescein acetoxymethyl ester (BCECF/AM)	Beyotime (Shanghai, China)	#S1006
DMEM medium	Thermo (Waltham, MA, USA)	#21,010,046
Radioimmunoprecipitation assay (RIPA)	Beyotime (Shanghai, China)	#P0013C
Matrigel	Beyotime (Shanghai, China)	#C0383
MCP-1 ELISA kit (Human)	Neobioscience (Shenzhen, China)	#H250107-113a
TRIzol reagent	Solarbio (Beijing, China)	#15,596,026
Trypsin-EDTA (m/V, 0.25%)	Thermo (Waltham, MA, USA)	#25,200,056
1,2-Bis(dimethylamino)ethane	Solarbio (Beijing, China)	#T8090
10% SDS solution	Solarbio (Beijing, China)	#S1010
1.5 M Tris-HCl Buffer solution	Solarbio (Beijing, China)	#T1010
1.0 M Tris-HCl Buffer solution	Solarbio (Beijing, China)	#T2010
Rabbit polyclonal antibody lgG Anti-VEGF	HUABIO (Hangzhou, China)	#ER30607
Recombinant Rabbit mAb Anti-HIF-1α LCoR Rabbit PolyAb	HUABIO (Hangzhou, China) Proteintech (Wuhan, China)	#HA722778 #14476-1-AP
GAPDH Polyclonal antibody	Proteintech (Wuhan, China)	#10494-1-AP
Phenylmethylsulfonyl fluoride	Solarbio (Beijing, China)	#P0100
Crystal Violet Staining solution	BKMAMLAB (Beijing, China)	#110,703,009
BeyoClick Edu-555 Cell Proliferation with Alexa Fluor 555	Beyotime (Shanghai, China)	#C0075S
PE anti-human CD206	Biolegend (San Diego, CA, USA)	#321,105
PE anti-human CD86	Biolegend (San Diego, CA, USA)	#305,441
2’,7’-bis-(2-carboxyethyl)-5-(and-6)-Carboxyfluorescein acetoxymethyl ester (BCECF/AM)	Beyotime (Shanghai, China)	#S1006

### Clinical human samples

A total of 10 glioma surgical specimens and corresponding peri-tumoral normal tissues (PTTs) were collected from patients who underwent primary surgery at the Department of Neurosurgery, Third People’s Hospital of Hainan Province, between 2023 and 2024. None of the patients received pre-operative radiotherapy or chemotherapy. Demographic characteristics were as follows: mean age 52.4 ± 8.7 years (range 35–68), 6 males and 4 females; World Health Organization (WHO) grade II (*n* = 3), grade III (*n* = 3), and grade IV (*n* = 3). Tissues were immediately immersed in RNAlater solution, incubated at 4 °C overnight to allow complete penetration, and then stored at -80 °C for long-term preservation. Written informed consent was obtained from all patients, and the study was approved by the Research Ethics Committee of the Third People’s Hospital of Hainan Province (approval No. LLKY2305107).

### TCGA analysis

The Cancer Genome Atlas (TCGA) glioma cohort (https://portal.gdc.cancer.gov) is a comprehensive public database that provides genomic and clinical data for various cancer types. It integrates RNA-seq expression profiles and corresponding clinical information, enabling the evaluation of gene expression levels and their association with patient prognosis. Using this database, we investigated the relationship between LCoR expression and overall survival in glioblastoma. Kaplan-Meier survival curves were generated, and statistical significance was determined using the log-rank test.

### Cell culture

Normal human astrocytes (NHAs, Procell, Wuhan, China) and glioblastoma cell lines (all authenticated by STR profiling by ATCC), including SNB-19, SF-295, LN229, U-87 MG, A172, and U251, were cultured in Dulbecco’s modified Eagle medium (DMEM) supplemented with 10% heat-inactivated fetal bovine serum (FBS) and 1% penicillin-streptomycin (PS). Human THP-1 monocytes and human umbilical vein endothelial cells (HUVECs) were cultured according to a previous protocol [13]. All cells were incubated at 37 °C under 5% CO₂.

### Plasmid construction and transfection

ShRNA oligo duplexes (50 nM, GenePharma) targeting LCoR mRNA were synthesized and cloned into the pLKO.1 vector (GenePharma, Shanghai). A non-targeting shRNA sequence was used as the negative control (KD-NC). Lentiviral particles were packaged in HEK293T cells, and SF-295 cells (MOI = 5, 8 µg/mL polybrene, 24 h) were infected with viral supernatant, followed by puromycin (2 µg/mL) selection to establish stable LCoR-knockdown (KD-LCoR) and control (KD-NC) cell lines. For LCoR overexpression, the LCoR coding sequence was cloned into a lentiviral vector (GenePharma, Shanghai), and an empty vector was used as the negative control (OE-NC). These constructs were transfected into SNB-19 cells (70% confluent, 5 × 10⁵/well, 6-well) using Lipofectamine 3000, followed by puromycin selection to establish LCoR-overexpressing (OE-LCoR) and control (OE-NC) cell lines.

### Colony formation assay

SF295 cell suspensions were seeded into culture dishes and treated with DNQ (4, 8, 16 nmol/L; 0.1% DMSO). After 7 days, colonies were fixed with 4% paraformaldehyde, stained with 0.1% crystal violet, and counted to calculate colony formation rates. The Bio-Rad ChemiDoc XRS + system was used to acquire images of the cell colonies, followed by quantification using ImageJ software.

### Scratch wound healing assay

Cells were grown to confluence in 6-well plates, and a sterile pipette tip was used to create linear scratches. Cells were treated with DNQ (4, 8, 16 nmol/L), and scratch closure was monitored at 0 h and 24 h. Migration was quantified using ImageJ.

### Cell viability assay

Cell viability was assessed using the cell counting kit-8 (CCK-8) assay. Briefly, cells were seeded in 96-well plates at a density of 1 × 10⁴ cells per well. After the respective treatments, 20 µL of CCK-8 solution was added to each well, followed by incubation at 37 °C for 4 h. The absorbance at 450 nm was measured using a BioTek microplate reader.

### Enzyme-linked immunosorbent assay (ELISA)

MCP-1 levels in cell supernatants were quantified using a commercial ELISA kit following the manufacturer’s protocol. First, the supernatant was added to a 96-well plate and incubated for 3 h, followed by successive incubation with biotinylated primary antibody for 2 h and streptavidin-HRP solution for 45 min. Next, the TMB development solution was added to each well and incubated for 10 min. Finally, absorbance at 450 nm was measured with a BioTek microplate reader.

### Establishment of a non-contact coculture system for glioma cells and macrophages​

A non-contact coculture model was established using Transwell inserts (polycarbonate membrane, pore size: 8.0 μm) to investigate the paracrine interactions between glioma cells and macrophages. Briefly, SNB19 and SF295 glioma cells, engineered to stably overexpress or knockdown the LCoR gene, were seeded in the lower chamber of the Transwell plate at a density of 2 × 10⁵ cells per well to precondition the tumor microenvironment. In the upper chamber, 4 × 10⁴ differentiated THP-1 macrophages were plated per insert. The two compartments were separated by a serum-free medium. Following 48 h of coculture, THP-1-derived macrophages were carefully harvested from the upper chambers for subsequent functional analyses.

### Transcriptome sequencing

SF295 cells were cultured to 90% confluence and then treated with 4 nmol/L DNQ for 6 h. After treatment, the cells were washed twice with pre-cooled PBS and lysed with Trizol reagent for total RNA extraction. Total RNA was also isolated from stable OE-NC and OE-LCoR SF295 cells. All RNA samples were subjected to high-throughput sequencing for comprehensive transcriptome profiling. Subsequent data analysis was performed using the free online Majorbio Cloud Platform (www.majorbio.com).

### Angiogenesis assay

HUVECs (2 × 10⁴ cells/well) were resuspended in conditioned medium and seeded onto Matrigel-coated 96-well plates. Vascular network formation was observed after 24 h, and junctions were quantified using ImageJ software [13].

### RNA extraction and qRT-PCR

Total RNA was extracted with TRIzol and reverse-transcribed into cDNA. qRT-PCR was performed using SYBR Green Master Mix on a Bio-Rad CFX Duet system. Primer sequences: LCoR (Forward: 5′-ATGCAGCG AATGATCCAACAA-3′; Reverse: 5′-CCAGAGGTGAGTCTTGGTCAG-3′), MCP-1 (Forward: 5′-CAGCCAGATGCAATCAATGCC-3′; Reverse: 5′-TGGAATCCTGAACCCACTTCT-3′), CCL20 (Forward: 5′-TGCTGTACCAAGAGTTTGCTC-3′; Reverse: 5′-CGCACACAGACAACTTTTTCTTT-3′), CSF-1 (Forward: 5′-AGCTGTGGCTACAGCGTTTCGAT-3′; Reverse: 5′-GCAATCAGGCTTGGTCACCACA-3′), HGF (Forward: 5′-GCTATCGGGGTAAAGACCTACA-3′; Reverse: 5′-CGTAGCGTACCTCTGGATTGC-3′), IL-3 (Forward: 5′-CCTCATGGCGCTTTTGTTGAC-3′; Reverse: 5′-TCTGGTTCTGGGTGATGTTGA-3′), IL-4 (Forward: 5′-CCAACTGCTTCCCCCCTCTG-3′; Reverse: 5′-TCTGTTACGGTCAACTCGGTG-3′), IL-8 (Forward: 5′-TCTGTTACGGTCAACTCGGTG-3′; Reverse: 5′-TTCTTTAAGCACTCCTTGGGCAAA-3′), IL-1β (Forward: 5′-ATGATGGCTTATTACAGTGGGCAA-3′; Reverse: 5′-GTCGGAGATTCGTAGCTGGA-3′), IFN-γ (Forward: 5′-TCGGTAACTGACTTGAATGTCCA-3′; Reverse: 5′-TCGCTTCCCTGTTTTAGCTGC-3′), CD86 (Forward: 5′-ATGATGGCTTATTACAGTGGGCAA-3′; Reverse: 5′-GGAAACGTCGTACAGTTCTGTG-3′), ARG-1 (Forward: 5′-ATGATGGCTTATTACAGTGGGCAA-3′; Reverse: 5′-GTCGGAGATTCGTAGCTGGA-3′), IL-10 (Forward: 5′-GACTTTAAGGGTTACCTGGGTTG-3′; Reverse: 5′-TCACATGCGCCTTGATGTCTG-3′), CD206 (Forward: 5′-TCCGGGTGCTGTTCTCCTA-3′; Reverse: 5′-CCAGTCTGTTTTTGATGGCACT-3′), and GAPDH (Forward: 5′-GTCTCCTCTGACTTCAACAGCG-3′; Reverse: 5′-ACCACCTGGTTGCTGTAGCCAA-3′).

### Western blot

Cells and tissues were lysed using RIPA lysis buffer containing 1 mM PMSF. Protein concentration was quantified utilizing BCA assay. Equal amounts of protein (20 µg) were separated by SDS-PAGE and transferred to PVDF membranes. Membranes were blocked with 5% skim milk at room temperature for 1 h, followed by overnight incubation with primary antibodies at 4 °C. Primary antibodies included: GAPDH (Proteintech, 10494-1-AP, 1:10000, Wuhan, China), LCoR (Proteintech, 14476-1-AP, 1:1000, Wuhan, China), VEGF (HUABIO, ER30607, 1:1000, Hangzhou, China), and HIF-1α(HUABIO, HA722778, 1:1000, Hangzhou, China). Membranes were then incubated with HRP-conjugated secondary antibodies (UpinBio, YP848537, 1:10000, Shenzhen, China) at room temperature for 2 h. Signals were detected with ECL Plus Western Blotting Reagent Pack (Tanon 4600 SF) and quantified with ImageJ software.

### EdU assay

Cell proliferation was assessed using a 5-ethynyl-2’-deoxyuridine (EdU) assay. Briefly, cells were pulsed with EdU for 2 h, then fixed with 4% paraformaldehyde and permeabilized with 0.5% Triton X-100. The incorporated EdU was subsequently labeled using a click reaction cocktail containing a fluorescent azide dye. Cell nuclei were counterstained with DAPI. Images were acquired using a fluorescence microscope (Leica DMi8), and the percentage of EdU-positive cells was quantified to determine the proliferation rate.

### Transwell assay

Cell migration and invasion were assessed using 8.0 μm pore-size Transwell inserts (Beyotime). For migration assays, 4 × 10⁴ cells suspended in serum-free DMEM were seeded into the upper chamber; for invasion assays, the inserts were pre-coated with Matrigel to reconstruct an extracellular matrix barrier. The lower chamber was filled with 600 µL complete medium containing indicated drugs to generate a chemotactic gradient. After 24 h incubation (37 °C, 5% CO₂), non-migrated/non-invaded cells on the upper surface were gently removed with a cotton swab. Cells on the underside were fixed with 4% paraformaldehyde for 15 min and stained with 0.1% crystal violet or labeled with 5 mmol/L BCECF/AM (#S1006, Beyotime). Five random high-power fields per membrane were captured under an inverted microscope, and the average number of migrated/invaded cells was used to calculate the migration or invasion rate.

### Flow cytometry

1 × 10⁶ THP-1 macrophages treated with conditioned medium were stained with CD86-FITC (#305441, Biolegend) and CD206-PE (#321105, Biolegend) antibodies (4 °C, 30 min). Cell populations were obtained and analyzed using a flow cytometer according to our previously established method [14].

### Immuno-histochemical staining (IHC) and hematoxylin-eosin (H&E) staining

The subcutaneous tumor and whole brain of tumor-bearing mice were isolated and fixed with 4% paraformaldehyde and embedded in paraffin. Then, 5 mm slices were prepared with a microtome, followed by deparaffinization, dehydration, and incubation in heat-mediated antigen retrieval sequentially. Subsequently, the IHC and H&E were performed according to our previous protocol [4].

### 3D tumor spheroid formation assay

5,000 SNB-19 single cells were suspended in 10 µL growth-factor-reduced Matrigel (1:1 v/v with complete DMEM) and plated as 3-droplet domes in 96-well ultra-low-attachment plates. After 10 min gelation at 37 °C, 100 µL complete medium was gently overlaid per well and refreshed every 4 days for 12 days. Live spheroids were imaged (×40 objective) on days 4, 8 and 12; volumes were calculated from orthogonal diameters (V = L × W² × 0.5) using ImageJ.

### In vivo xenograft studies

#### Animals

Four-week-old female BALB/c nude mice were purchased from Beijing Charles River Laboratory Animal Technology Co., Ltd (Beijing, China). The mice were housed in a specific pathogen-free (SPF) animal room with a 12-h light/12-h dark cycle and free access to food and water. All animal experiments were approved by the Animal Ethics Committee of Guizhou Medical University (Approval No.: 2303299) and conducted in accordance with institutional guidelines.

#### Cell preparation

OE-LCoR SNB-19 cells, KD-LCoR SF295 and their corresponding control cells were harvested during the logarithmic growth phase. The cells were washed, counted, and resuspended in a sterile mixture of phosphate-buffered saline and growth factor-reduced Matrigel (1:1) to a specific concentration for injection. Cell viability, confirmed by Trypan Blue exclusion to be > 95%, was ensured prior to implantation.

#### Subcutaneous xenograft model

For the subcutaneous model, female BALB/c nude mice (4 weeks old, *n* = 6) received a single subcutaneous injection of 1 × 10⁷ cells in 100 µL of suspension into the right armpit. Tumor growth was monitored twice weekly by measuring the tumor dimensions using a digital caliper. Tumor volume (V) was calculated using the formula: V = (Length × Width²) /2. The mice were euthanized when the tumor volume reached approximately 1000 mm³, and the tumors were harvested for further analysis.

#### Orthotopic xenograft model

The intracranial orthotopic model was replicated based on our previously established protocol [4]. Animal well-being and neurological symptoms were monitored daily. When obvious neurological symptoms appeared, the whole brains of tumor-bearing mice were harvested to assess tumor growth.

#### Euthanasia method

Mice were euthanized by cervical dislocation following deep anesthesia induced by intraperitoneal injection of amobarbital sodium at a dose of 150–200 mg/kg. Loss of consciousness and absence of response to painful stimuli were confirmed prior to euthanasia.

### Statistical analysis

All data are presented as mean±standard deviation (SD). “n = 3” indicates three independent experiments performed on different days with separately prepared cell cultures. For animal studies, “n” refers to the number of individual mice per group. Data normality was verified using the Shapiro-Wilk test. Comparisons between two groups were performed using a two-tailed Student’s t-test, while comparisons among three or more groups were conducted using one-way ANOVA followed by Dunnett’s post hoc test. A *P* value < 0.05 was considered statistically significant. Data analysis was performed using GraphPad Prism software (version 10).

## Results

### Reduced LCoR expression correlates negatively with glioma pathological grading

**Fig. 1:**
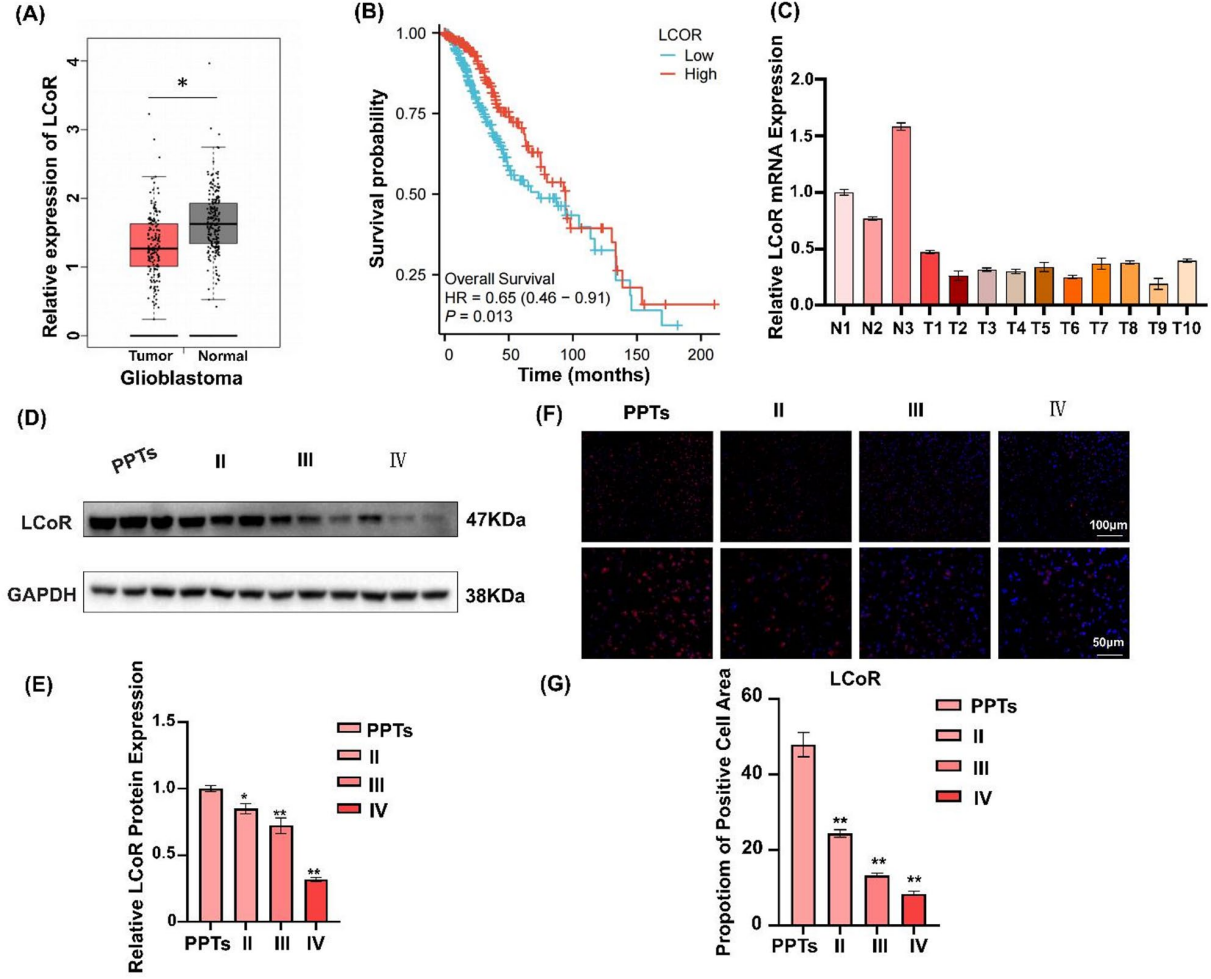
Reduced LCoR expression correlates negatively with glioma pathological grading. (**A**) Analysis based on the TCGA public database shows significantly downregulated LCoR expression in glioblastoma tissues. (**B**) Kaplan-Meier survival curves indicate that patients with high LCoR expression have a longer overall survival. (**C**-**D**) mRNA and protein expression levels of LCoR in peri-tumoral normal tissues (PTTs) and glioma tissues (WHO grades Ⅱ, Ⅲ, Ⅳ) were detected by qRT-PCR and Western blot (*n* = 3). (**E**, **F**) Immunofluorescence staining analysis of LCoR expression in PTTs and glioma tissues (WHO grades Ⅱ, Ⅲ, Ⅳ) (*n* = 3). * *P* < 0.05, ** *P* < 0.01 vs. the PPTs group

The Cancer Genome Atlas (TCGA) is a public database that provides gene expression profiles and clinical data for various cancers [15]. TCGA organizes the expression values of each searchable gene in different tumor samples, which can calculate the expression level of a gene in a specific type of tumor and can also analyze the relationship between genes and tumor prognosis, co-expression between genes, etc. Using this method, we searched for the relationship between LCoR and glioblastoma. The results showed that the expression level of the LCoR gene in glioblastoma tissue was significantly lower than that in normal tissue, and the expression of the LCoR gene was positively correlated with the overall survival of patients (Figs. 1A & B). This aroused our curiosity, and we further compared the expression levels of LCoR in glioma tissue and peri-tumor tissues (PTTs). Results from qRT-PCR and Western blot showed that the mRNA and protein levels of LCoR in glioma tissue were significantly lower than those in PTTs (Figs. 1C-E), which was further confirmed by the immunohistochemistry (IHC) assay (Figs. 1F&G).

### LCoR suppresses the malignant phenotypes of glioblastoma by inhibiting the expression of VEGF and HIF-1α

**Fig. 2:**
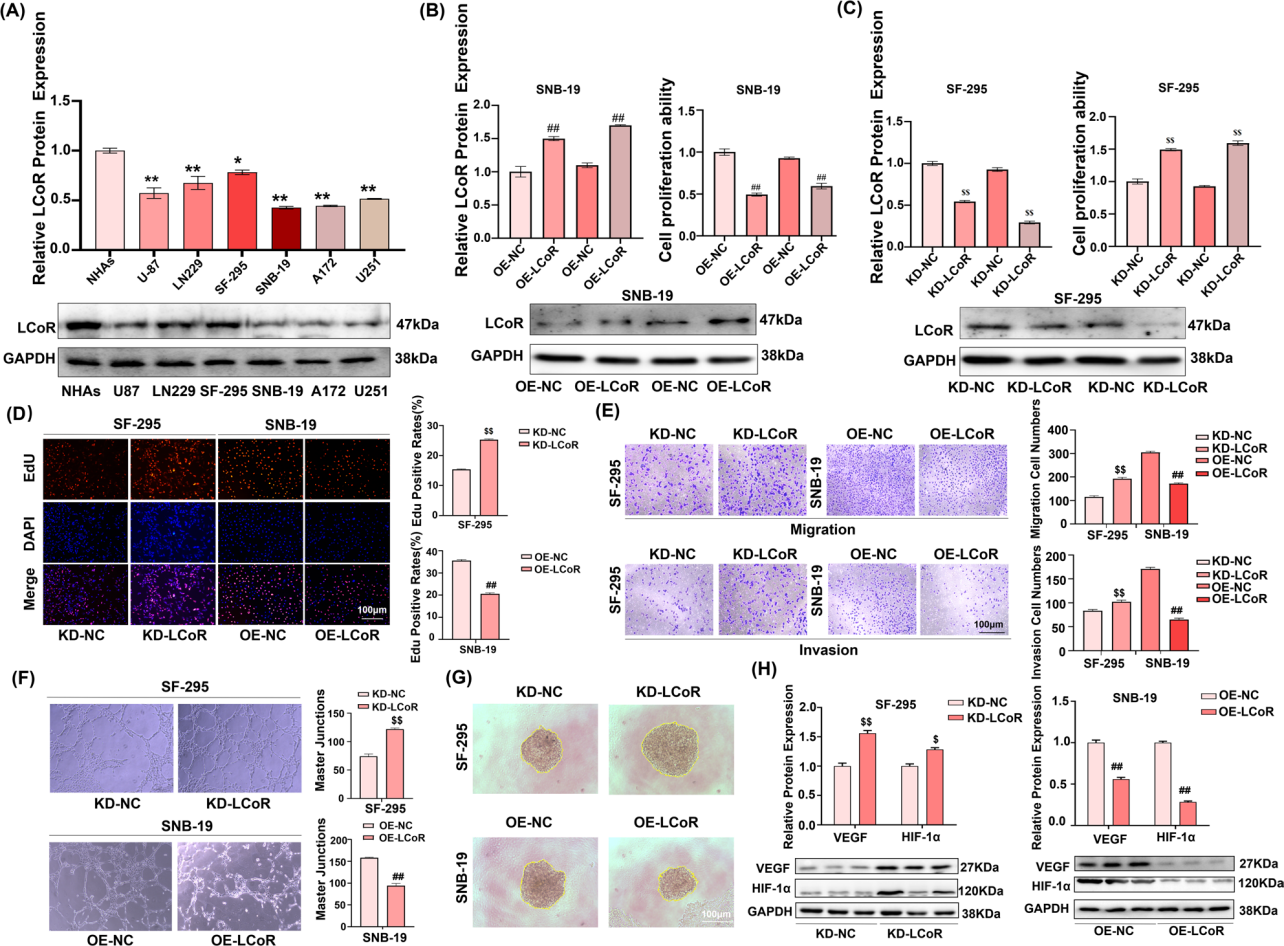
LCoR suppresses the malignant phenotype of glioblastoma by inhibiting VEGF and HIF-1α expression. (**A**) Western blot reveals the basal expression levels of LCoR in normal human astrocytes (NHAs) and glioma cell lines (*n* = 3). (**B**) Western blot and CCK8 assays show reduced proliferation activity in SNB-19 cells after LCoR-overexpression (OE-LCoR) via plasmid transfection (*n* = 3). (**C**) Western blot and CCK8 assays demonstrate increased proliferation activity in SF-295 cells after LCoR-knockdown (KD-LCoR) via ShRNA (*n* = 3). (**D**) EdU staining evaluates DNA synthesis capacity in KD-NC, KD-LCoR, OE-NC, and OE-LCoR groups (*n* = 3). (**E**) Transwell assay analyzes the effect of LCoR regulation on glioma cell migration and invasion (*n* = 3). (**F**, **G**) Angiogenesis and 3D tumor spheroid formation assays reveal LCoR’s regulatory role in vasculogenic mimicry and tumor spheroid formation (*n* = 3). (**H**) Western blot detects expression levels of angiogenesis-related markers VEGF and HIF-1α (*n* = 3). *P < 0.05 and **P < 0.01 vs. the NHAs group; ^##^*P* < 0.01 vs. the OE-NC group; ^$^*P* < 0.05 and ^$$^*P* < 0.01 vs. the KD-NC group

The functional consequences of regulating LCoR expression in human glioblastoma cell lines were investigated. First, results from Western blot showed that the expression of LCoR in human astrocytes (NAHs) was higher than that of glioblastoma cells (Fig. 2A). To assess the effects of LCoR gain- and loss-of-function, we established OE-LCoR and OE-NC cell lines in SNB-19 cells, as well as KD-LCoR and KD-NC cell lines in SF-295 cells, as described in the Methods section (2.5). CCK-8 assay showed that OE-LCoR decreased the proliferation ability of SNB-19 cells, but KD-LCoR increased the proliferation ability of SF-295 (Figs. 2B&C). Similarly, the 5-ethynyl-2′-deoxyuridine (EdU) assays indicated that OE-LCoR impaired the proliferation of glioblastoma cells, whereas KD-LCoR accelerated cell growth (Fig. 2D). The transwell assay showed that OE-LCoR suppressed the migration and invasion of glioblastoma cells, while KD-LCoR led to opposite effects (Fig. 2E). Meanwhile, LCoR inhibited angiogenesis and tumor sphere growth (Figs. 2F&G). In addition, LCoR reduced the expression of VEGF and HIF-1α (Fig. 2H), both of which are widely reported to promote the malignant progression of glioma. These findings indicated that the LCoR gene has a suppressive effect on the growth of glioblastoma cells, and OE-LCoR can inhibit malignant phenotypes of glioblastoma via reducing the expression of VEGF and HIF-1α in vitro.

### LCoR reduces macrophage migration toward glioblastoma cells and inhibits M2 polarization in vitro

**Fig. 3:**
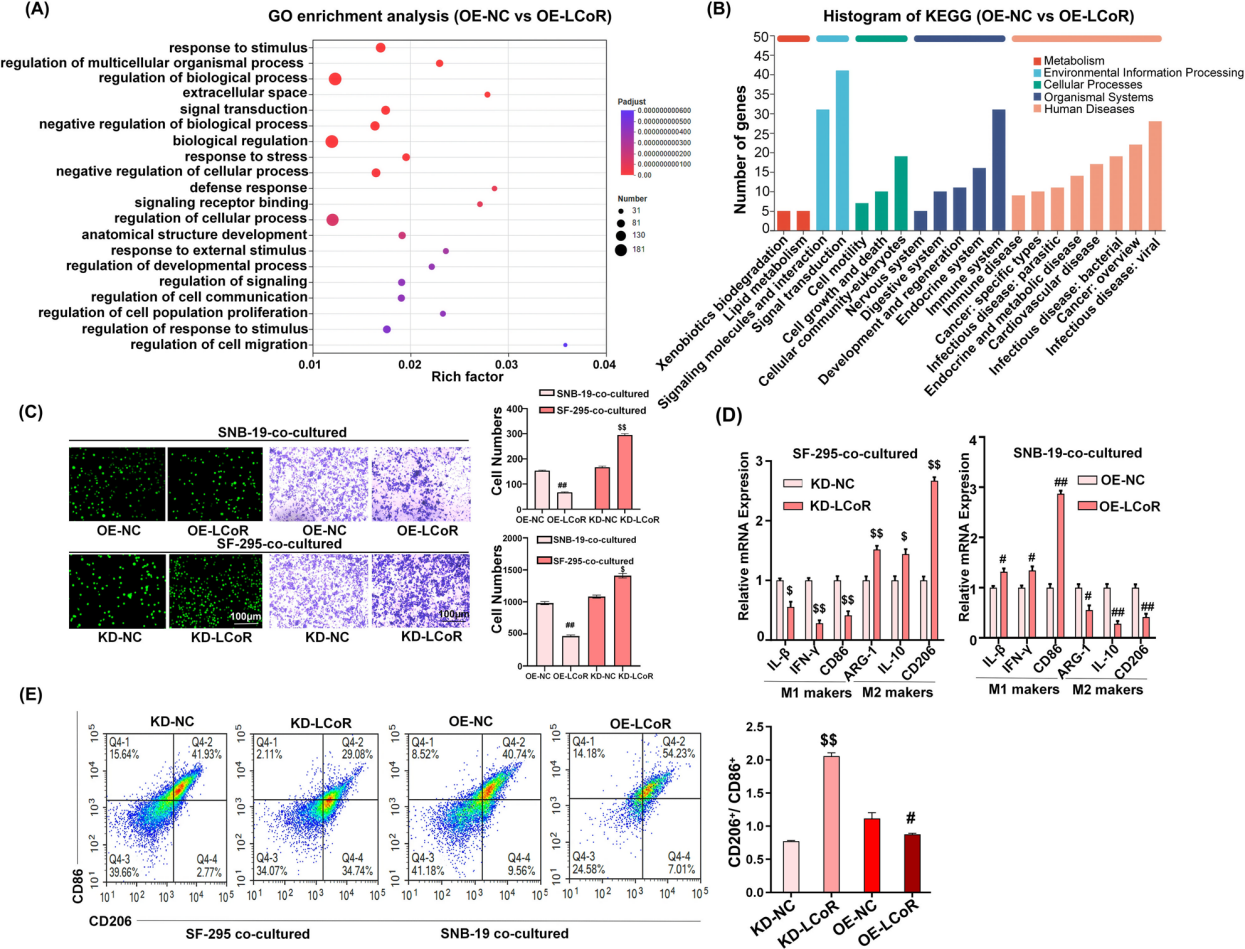
LCoR reduces macrophage migration toward tumor cells and inhibits M2 polarization of macrophages in vitro. (**A**, **B**) GO enrichment analysis of transcriptome sequencing data suggests LCoR regulates proliferation-migration-related genes, while KEGG analysis shows its involvement in immune system-related pathways. (**C**) A Transwell co-culture model demonstrates an inverse correlation between LCoR expression in glioma cells and migration ability of THP-1 monocytes (left, fluorescently labeled) or THP-1 macrophages (right, crystal violet-stained) (*n* = 3). (**D**) qRT-PCR detects M1/M2 polarization markers in THP-1 macrophages within the co-culture system (*n* = 3). (**E**) Flow cytometry quantifies the proportion of CD86⁺ and CD206⁺ macrophage subsets (*n* = 3). ^#^*P* < 0.05 and ^##^*P* < 0.01 vs. the OE-NC group; ^$^*P* < 0.05 and ^$$^*P* < 0.01 vs. the KD-NC group

GO enrichment and KEGG analysis based on the data of transcriptome sequencing showed that, in addition to regulating the proliferation and migration of tumor cells, LCoR also regulates the immune system (Fig. 3A&B). Tumor-associated macrophages (TAMs) are the most important cells involved in immune regulation of the tumor microenvironment [16]. TAMs are derived from blood monocytes and tissue-resident macrophages. To explore whether LCoR was involved in the immune regulation of TAMs in the glioma microenvironment, an in vitro coculture model was constructed based on Transwell chambers. The Transwell migration assay showed that, compared to the OE-NC group, OE-LCoR glioma cells weakened the migration of THP-1 monocytes and PMA-induced THP-1 macrophages, while KD-LCoR promoted this process (Fig. 3C). Furthermore, qRT-PCR results showed that knocking down LCoR in glioblastoma cells induced M2 polarization of THP-1 macrophages, while overexpressing LCoR promoted M1 polarization (Fig. 3D). In addition, flow cytometry analysis showed that the ratio of CD206^+^/CD86^+^ cells was significantly upregulated in the KD-LCoR group, but downregulated in the OE-LCoR group (Fig. 3E). Altogether, these results indicated that LCoR reduced the recruitment of TAMs and the M2 polarization.

### LCoR suppresses macrophage migration toward glioblastoma cells and M2 polarization by reducing chemokine secretion

**Fig. 4:**
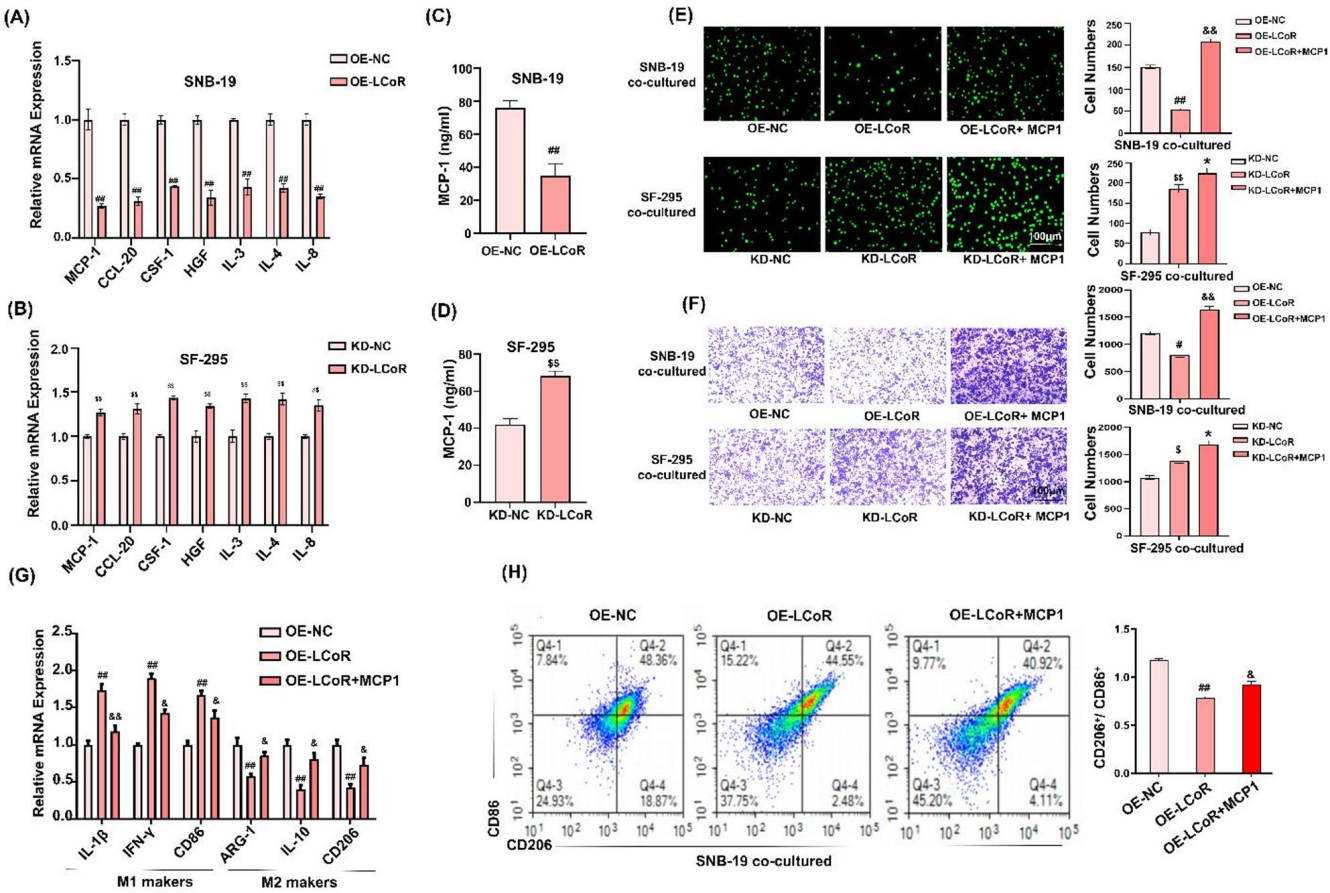
LCoR suppresses macrophage migration toward glioma cells and M2 macrophage polarization by reducing chemokine secretion. (**A**, **B**) qRT-PCR detect mRNA level of chemokines in OE-LCoR (SNB-19) and KD-LCoR (SF-295) glioma cells (*n* = 3). (**C**, **D**) ELISA quantifies MCP-1 secretion levels in cell supernatants (*n* = 3). (**E**, **F**) Transwell assay shows that exogenous MCP-1 (40 ng/mL) reverses the regulatory effect of OE-LCoR on migration of THP-1 monocytes (fluorescently labeled) and macrophages (crystal violet-stained) (*n* = 3). (**G**) qRT-PCR reveals that exogenous MCP-1 (40 ng/mL) alters M1/M2 polarization marker expression in THP-1 macrophages (*n* = 3). (**H**) Flow cytometry demonstrates that exogenous MCP-1 (40 ng/mL) modulates M1/M2 macrophage ratios (*n* = 3). ^#^*P* < 0.05 and ^##^*P* < 0.01 vs. the OE-NC group; ^&^*P* < 0.05 and ^&&^*P* < 0.01 vs. the OE-LCoR group; ^$^*P* < 0.05 and ^$$^*P* < 0.01 vs. the KD-NC group; *P < 0.05 vs. the KD-LCoR group

It has been reported that the infiltration of TAMs is related to chemokines, such as MCP-1, IL-8, and CSF1 [17]. Thus, we investigated whether LCoR inhibited glioblastoma progression and TAMs infiltration via regulating the expression of chemokines. Results from qRT-PCR demonstrated OE-LCoR decreased the mRNA levels of TAMs infiltration-associated chemokines, especially reduced the expression of MCP-1 (Fig. 4A). In contrast, MCP-1 was increased in KD-LCoR SF-295 cells (Fig. 4B). More interesting, consistent changes of MCP-1 were detected in the supernatants of OE-LCoR SNB-19 or KD-LCoR SF-295 cells (Figs. 4C&D). Meanwhile, the Transwell coculture assay showed that exogenous supplementation of MCP-1 could partly reverse the migration of THP-1 monocytes or macrophages in OE-LCoR SNB-19 (Figs. 4E&F). Additionally, LCoR-mediated reduction of M2 polarization of macrophages was partly reversed by the exogenous supplementation of MCP-1 (Figs. 4G&H). These data indicated that LCoR could suppress macrophage migration toward glioblastoma cells and M2 polarization by reducing chemokines secretion, especially the regulation of MCP-1.

### LCoR inhibits glioblastoma growth by suppressing angiogenesis and M2 macrophage polarization

**Fig. 5:**
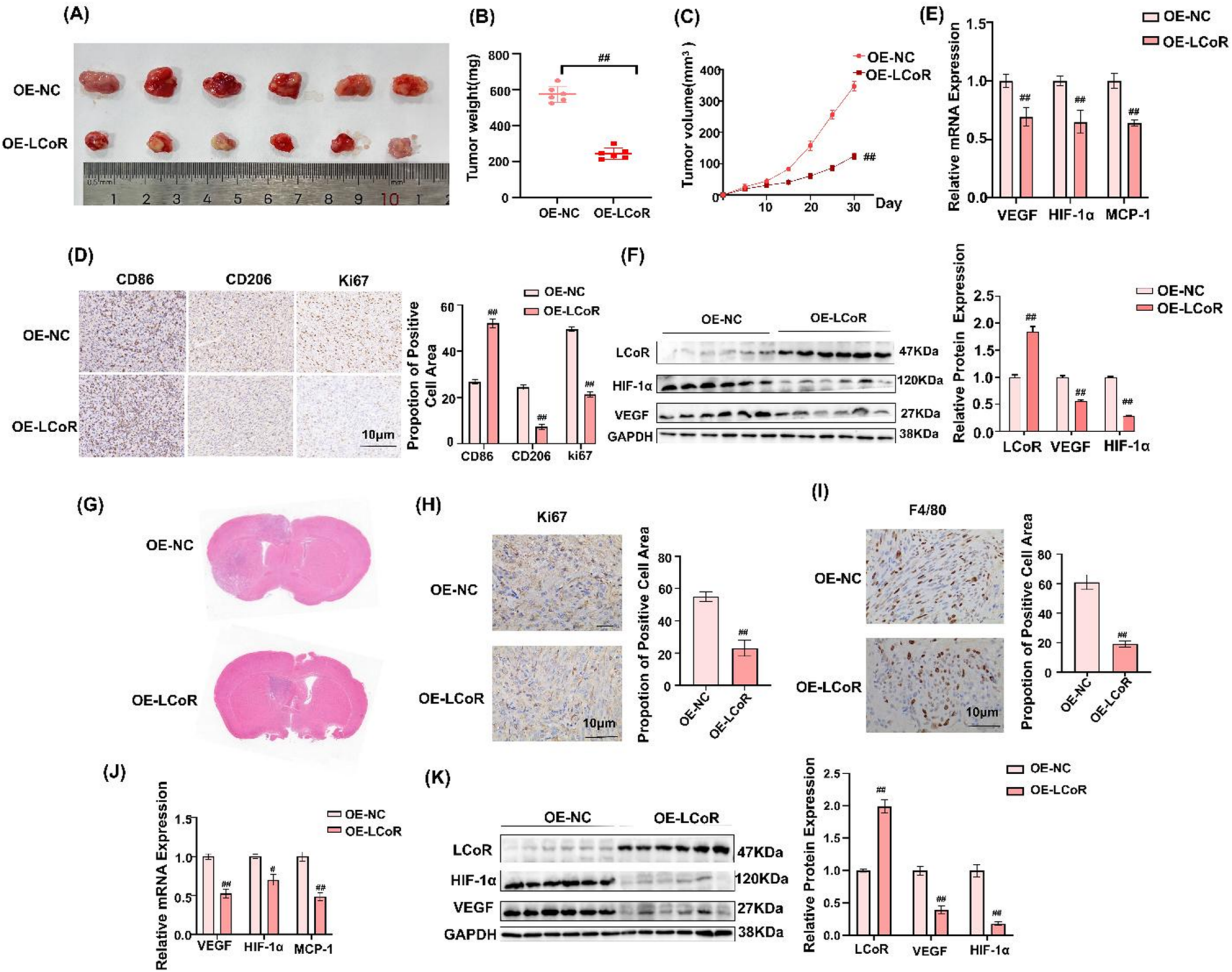
LCoR inhibits glioblastoma growth by suppressing angiogenesis and M2 macrophage polarization. (**A**) Subcutaneous xenografts show reduced tumor volume and weight in the OE-LCoR group (*n* = 6). (**B**, **C**) Tumor growth curves and final tumor weights (*n* = 6). (**D**) Immunohistochemistry quantifies Ki-67, CD86⁺, and CD206⁺ cell densities (*n* = 3). (**E**, **F**) qRT-PCR and Western blot confirm OE-LCoR downregulates VEGF, HIF-1α, and MCP-1 in subcutaneous tumors (*n* = 6). (**G**) H&E staining shows that OE-LCoR suppresses intracranial glioblastoma growth (*n* = 5). (**H**, **I**) Immunohistochemistry detects Ki-67 (proliferation marker) and F4/80 (macrophage marker) density in tumor tissues (*n* = 6). (**J**-**K**) qRT-PCR and Western blot reveal OE-LCoR reduces VEGF, HIF-1α, and MCP-1 expression in intracranial xenografts (*n* = 5). ^#^*P* < 0.05 and ^##^*P* < 0.01 vs. the OE-NC group

To investigate the role of LCoR in glioblastoma progression in vivo, a subcutaneous tumor-bearing model was established using OE-NC or OE-LCoR SNB-19 cells. As shown in Figs. 5A-C, OE-LCoR significantly inhibited the development of the subcutaneous tumor. Ki67 staining showed that OE-LCoR suppressed the proliferation of SNB-19 cells in tumor tissue. The immunohistochemical assay showed that OE-LCoR decreased the CD86-positive cells but increased the CD206-positive cells when compared to the OE-NC group (Fig. 5D). Furthermore, Western blot and qPCR showed that OE-LCoR decreased the expression of angiogenesis-related proteins (VEGF, HIF-1α) and chemokine (MCP-1) (Figs. 5E&F). These results were similarly confirmed in an orthotopic tumor model in nude balb/c mice (Figs. 5G-K). Collectively, these data suggested that OE-LCoR limits glioblastoma progression in vivo, potentially through dual mechanisms of angiogenesis suppression and macrophage M2 polarization blockade.

### LCoR upregulator suppresses glioblastoma growth via up-regulating LCoR expression

**Fig. 6:**
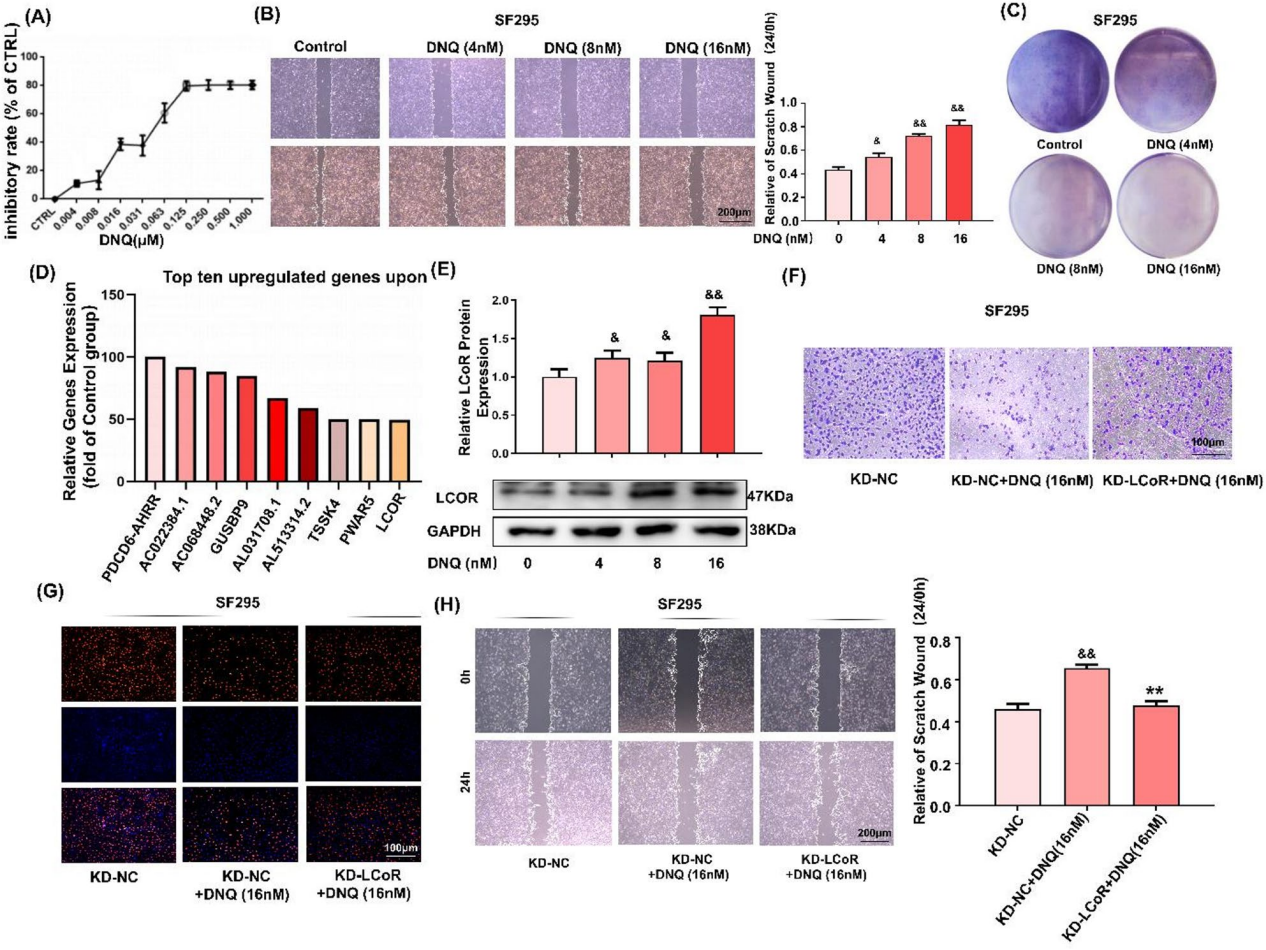
LCoR upregulator suppresses glioblastoma growth by upregulating LCoR expression. (**A**) MTT assay determines the inhibitory effect of deoxynyboquinone (DNQ) on glioma cells (*n* = 3). (**B**) Scratch assay evaluates SF295 cell migration under different DNQ concentrations (4, 8, 16 nmol/L; *n* = 3). (**C**) Colony formation assay analyzes DNQ’s inhibitory effect on SF295 cells (*n* = 3). (**D**) Transcriptome sequencing identified LCoR as one of the top 10 upregulated genes following DNQ treatment (*n* = 3). (**E**) Western blot confirms DNQ upregulates LCoR in a dose-dependent manner (*n* = 3). (**F**-**H**) Transwell, EdU, and scratch assays show KD-LCoR reverses DNQ’s inhibitory effects on SF295 cell proliferation and migration (*n* = 3). ^&^*P* < 0.05 and ^&&^*P <* 0.01 vs. the KD-NC group; **P < 0.01 vs. the KD-NC + DNQ (16 nM) group

The above research results suggest that LCoR is lowly expressed in glioblastoma, while its overexpression inhibits glioblastoma. Therefore, small molecules or gene therapy strategies that enhance LCoR activity may counteract its loss and improve patient outcomes. Deoxynyboquinone (DNQ), a natural small molecule, was discovered in marine actinomycetes in 2011 [18]. It has been shown to alter inhibitory activity on the proliferation of various tumor cells, but its anti-tumor effects on glioblastoma have not yet been fully elucidated. In this study, we found that DNQ inhibited the proliferation (Fig. 6A), migration (Fig. 6B), and cloning ability (Fig. 6C) of glioblastoma cells. Transcriptome sequencing results showed that DNQ upregulated the LCoR gene by 49.1-fold, ranking among the top 10 upregulated genes (Fig. 6D). Results from Western blot showed that DNQ dose-dependently upregulated the expression of LCoR (Fig. 6E). These results suggested that DNQ limits the growth of glioblastoma at least in part by upregulating LCoR expression. Therefore, we evaluated the anti-tumor activity of DNQ in the KD-LCoR SF-295 cells. As expected, the suppression effects of DNQ on the proliferation and migration of glioblastoma were greatly weakened when the LCoR was knocked down (Figs. 6F&H), which indicates that DNQ, acting as an LCoR upregulator, suppressed glioblastoma growth at least partially through increasing LCoR expression.

### LCoR upregulator suppresses angiogenesis and macrophage infiltration via MCP-1 downregulation

**Fig. 7:**
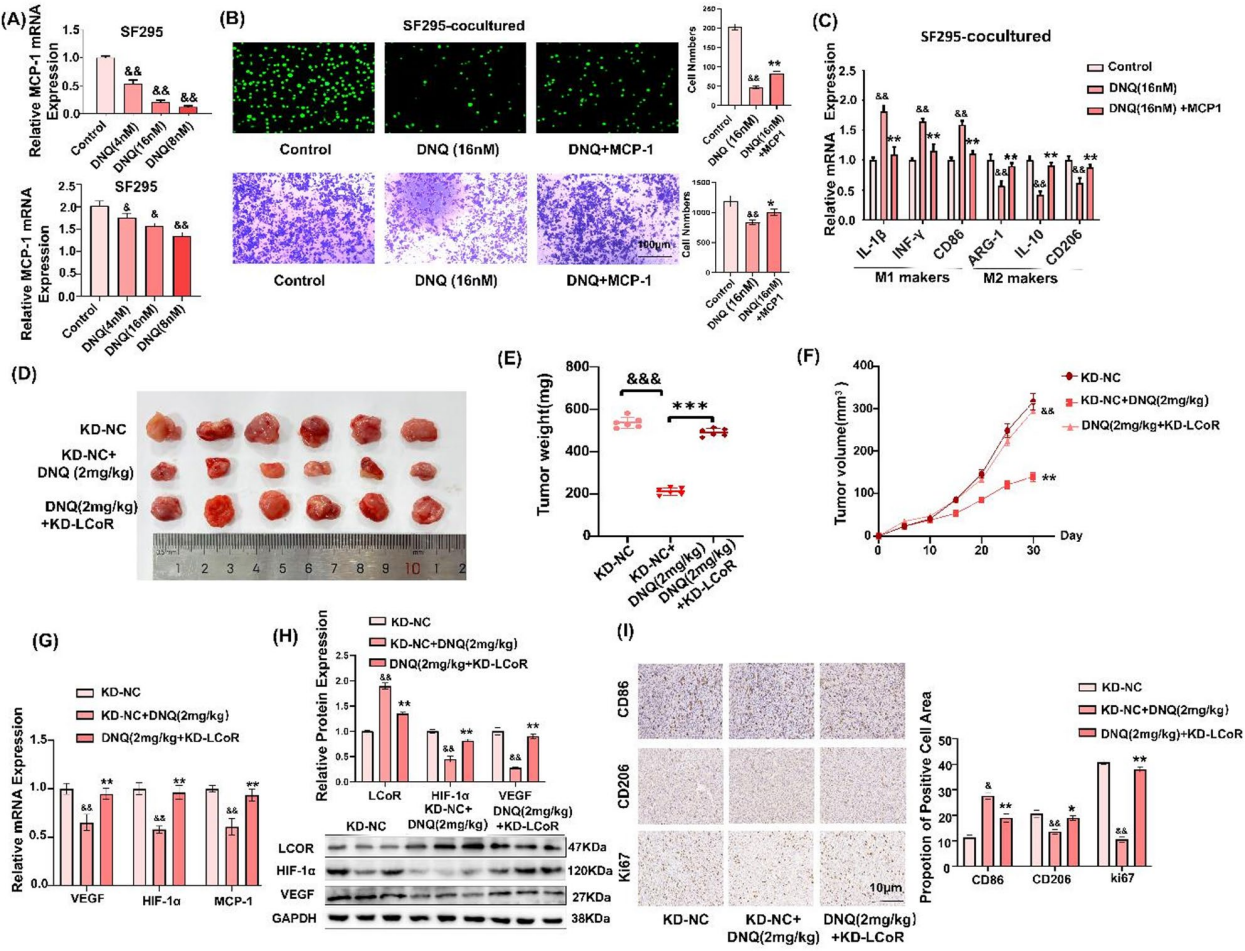
LCoR upregulator suppresses MCP-1 and macrophage infiltration via LCoR upregulation in mice. (**A**) qRT-PCR and ELISA show DNQ reduces MCP-1 mRNA and protein levels in SF295 cells (*n* = 3). (**B**) Transwell assays demonstrate exogenous MCP-1 (40 ng/mL) reverses DNQ’s suppression of THP-1 monocyte/macrophage migration (*n* = 3). (**C**) qRT-PCR reveals exogenous MCP-1 (40 ng/mL) counteracts DNQ’s regulation of M1/M2 polarization (*n* = 3). (**D**-**F**) Subcutaneous xenografts confirm DNQ (2 mg/kg) inhibits tumor growth, an effect attenuated by KD-LCoR (*n* = 3). (**G**-**H**) qRT-PCR and Western blot show DNQ downregulates VEGF, HIF-1α and MCP-1, reversed by KD-LCoR (*n* = 3). (**I**) Immunohistochemistry quantifies Ki-67, CD86⁺, and CD206⁺ cell densities in tummor tissues (*n* = 3). ^&^*P* < 0.05 ^&&^*P* < 0.01 and ^&&&^*P* < 0.001 vs. the KD-NC group; *P < 0.05, **P < 0.01 and ***P < 0.001 vs. the KD-NC + DNQ (2 mg/kg) group

Then, we further investigated the effects of DNQ as an LCoR upregulator on MCP-1 and macrophage infiltration. Results from qPCR and ELISA demonstrated that DNQ inhibited the mRNA levels of MCP-1 in the SF295 cells and reduced the secretion of MCP-1 in the supernatant (Fig. 7A). Moreover, when we co-cultured THP-1 monocytes or macrophages with the supernatant of DNQ-treated SF295 cells, the migration ability of these cells was decreased, and the expression of M2 markers (ARG-1, IL-10, and CD206) was also decreased; these effects were partly reversed by the exogenous supplementation of MCP-1 (Figs. 7B&C). Importantly, DNQ inhibited the growth of subcutaneous tumors and angiogenesis (Figs. 7D-F), decreased expression of MCP-1 and the infiltration of M2 macrophages (Figs. 7G-I), which were reversed in the KD-LCoR group. The above results suggest that DNQ, acting as an LCoR upregulator, suppresses angiogenesis and macrophage infiltration by downregulating MCP-1, thereby blocking glioblastoma progression.

## Discussion

Glioblastoma progression is critically driven by the dynamic interplay between tumor-associated macrophages (TAMs) and tumor-angiogenesis, forming a self-reinforcing loop within the immunosuppressive tumor microenvironment (TME) [19]. TAMs, which constitute 30–50% of the glioblastoma mass and predominantly exhibit an M2-polarized phenotype, secrete pro-angiogenic factors (e.g., VEGF). These factors directly stimulate endothelial cell proliferation, degrade the extracellular matrix, and promote the formation of disorganized, leaky blood vessels that exacerbate hypoxia and inflammation [20]. Hypoxia further amplifies TAMs activation via HIF-1α, perpetuating a cycle of aberrant angiogenesis and immune suppression. Concurrently, glioblastoma-associated endothelial cells recruit circulating monocytes by expressing chemokines like MCP-1/CCL2 and adhesion molecules (e.g., VCAM-1), which differentiate into TAMs skewed toward M2 polarization by endothelial-derived TGF-β and prostaglandin E2 (PGE2) [21]. This bidirectional crosstalk not only fuels tumor growth and invasion but also enhances therapy resistance by promoting vascular co-option and glioblastoma stem cell survival. Disrupting the TAM-angiogenesis synergy represents a promising avenue to mitigate glioblastoma’s aggressiveness and improve clinical outcomes.

In this study, our data demonstrate that LCoR expression is significantly reduced in glioblastoma compared to normal brain tissues and peritumoral regions, correlating positively with patient survival. This aligns with emerging evidence that nuclear receptor corepressors, such as N-CoR and SMRT, often function as tumor suppressors by repressing oncogenic transcriptional programs. The progressive loss of LCoR across glioma grades suggests its involvement in malignant transformation. Mechanistically, KD-LCoR enhanced proliferation, migration, and invasion in glioblastoma cells, whereas its overexpression exerted opposite effects. These observations are consistent with studies showing that corepressors modulate cancer cell plasticity by interacting with histone deacetylases (HDACs) and chromatin remodelers to silence pro-tumorigenic genes [22]. Importantly, our identification of VEGF and HIF-1α as downstream targets of LCoR provides a direct link between LCoR deficiency and the hypervascular phenotype of glioblastoma. VEGF, a key driver of angiogenesis, is transcriptionally regulated by HIF-1α under hypoxic conditions [23]. By suppressing both VEGF and HIF-1α, LCoR disrupts the hypoxia-angiogenesis feedback loop that fuels glioblastoma aggression. This dual inhibition is particularly relevant given the limited efficacy of single-agent anti-VEGF therapies like bevacizumab, which often trigger compensatory HIF-1α upregulation [24]. Our findings suggest that LCoR restoration could overcome such resistance by simultaneously targeting multiple nodes of angiogenic signaling.

A novel aspect of our work is the discovery that LCoR governs immune evasion in glioblastoma by modulating chemokine secretion, particularly monocyte chemoattractant protein-1 (MCP-1/CCL2). TAMs, which constitute up to 50% of the glioblastoma mass, are predominantly polarized to an immunosuppressive M2 phenotype, promoting tumor growth, angiogenesis, and therapy resistance [5]. We disclosed that LCoR suppresses monocyte recruitment and M2 polarization by downregulating MCP-1, a chemokine central to TAM infiltration [25]. This mechanism is reinforced by our in vitro and in vivo models: LCoR-overexpressing tumors exhibited reduced F4/80^+^ TAM infiltration and lower CD206^+^/CD86^+^ M2/M1 ratios. F4/80 is a widely used murine pan-macrophage membrane glycoprotein marker, its positive staining indicates the presence and infiltration of total macrophages in tumor tissues. The ability of exogenous MCP-1 to partially rescue TAM migration and M2 polarization in LCoR-overexpressing cells underscores the specificity of this axis. These results align with recent studies implicating MCP-1 as a master regulator of TAM dynamics in glioblastoma [26].

Previous studies have shown that RIP140 is an essential molecule for LCoR to exert its inhibitory effects on gene expression and cell proliferation [11, 27, 28]. The mRNA expression levels of RIP140 and LCoR are significantly positively correlated in biopsy tissues from breast and gastrointestinal cancers, and they can form a complex. Their interaction depends on the HTH domain of LCoR and the N-terminal and C-terminal regions of RIP140 [11]. Another study has found that overexpression of RIP140 in hepatocellular carcinoma inhibits M2 polarization of TAMs, promotes their conversion to the M1 phenotype, suppresses hepatocellular carcinoma cell invasion, induces apoptosis, and inhibits tumor growth. The mechanism is associated with the inhibition of the NF-κB/IL-6 signaling axis activation in TAMs [29], and NF-κB regulates the transcription of M1 polarization genes, including MCP-1 [30]. Therefore, LCoR likely regulates MCP-1 transcription through intermediate signaling pathways such as NF-κB.

Small molecule deoxynyboquinone (DNQ) [31], a marine-derived compound, upregulated LCoR expression by 49-fold in transcriptomic analyses and dose-dependently inhibited glioblastoma proliferation, migration, and clonogenicity. Crucially, DNQ’s anti-tumor effects were significantly attenuated in KD-LCoR cells, confirming LCoR as its primary mechanistic target. DNQ also phenocopied the effects of LCoR-overexpression in vivo, suppressing angiogenesis, reducing MCP-1 levels, and diminishing M2 TAM infiltration. These findings position DNQ as an LCoR upregulator with dual anti-angiogenic and immunomodulatory properties. It is important to note that although DNQ significantly upregulates LCoR expression at both mRNA and protein levels, the precise mechanism by which DNQ exerts this effect remains to be elucidated. Previous studies have shown that DNQ induces cancer cell death through NQO1-dependent ROS production, involving futile redox cycling and oxidative stress [31, 32]. Thus, NQO1-dependent oxidative stress pathways and LCoR-mediated pathways may act synergistically, a possibility worthy of further investigation.

Notably, DNQ’s ability to inhibit MCP-1 secretion and TAMs recruitment addresses a major limitation of current anti-angiogenic therapies. While drugs like bevacizumab transiently normalize vasculature, they often exacerbate immunosuppression by increasing hypoxia and TAMs infiltration [33]. In contrast, DNQ’s coordinated suppression of VEGF and MCP-1 may achieve vascular normalization without amplifying immune evasion. This aligns with the emerging concept of “vascular-immune crosstalk” in glioblastoma therapy, where combined targeting of angiogenesis and TAMs yields synergistic benefits [34].

In summary, our study establishes LCoR as a pivotal tumor suppressor in glioblastoma, operating at the intersection of angiogenesis and immune evasion. By downregulating VEGF/HIF-1α and MCP-1, LCoR disrupts the synergistic interplay between aberrant vasculature and immunosuppressive TAMs, offering a novel therapeutic axis. The identification of DNQ as a small molecule that upregulates LCoR expression underscores the translational potential of targeting this pathway. Future work should focus on optimizing LCoR-based therapies and integrating them into multimodal treatment regimens to combat this recalcitrant malignancy. Furthermore, although our study provides compelling evidence for the tumor‑suppressive role of LCoR, certain limitations should be noted. First, rescue experiments were not included in the LCoR knockdown assays to further confirm the specificity of the observed effects. Second, while the experimental models used are appropriate for initial investigation, validation in primary glioblastoma cells or more representative macrophage models would further support the robustness of the conclusions. Third, the specificity of DNQ for LCoR requires further validation. Fourth, the clinical sample size (*n* = 10) is relatively small. Although the results were consistent across all samples, validation in a larger independent cohort is needed to confirm the clinical relevance of LCoR. We plan to address these limitations in future studies by performing rescue experiments, using more clinically relevant models, and expanding the clinical sample set.

## Supplementary Information

Below is the link to the electronic supplementary material.

Supplementary Material 1

## Data Availability

Data will be made available on request.
