# Supplementary material for: Ligand-dependent corepressor suppresses angiogenesis and macrophage infiltration in gliobl astoma microenvironment via MCP-1 down-regulation
Source: BMC Biotechnol. 2026 May 18;26:88. doi: 10.1186/s12896-026-01166-6 (PMC13371665; doi:10.1186/s12896-026-01166-6)

Figure 1D(LCoR)original western blot for three repeats

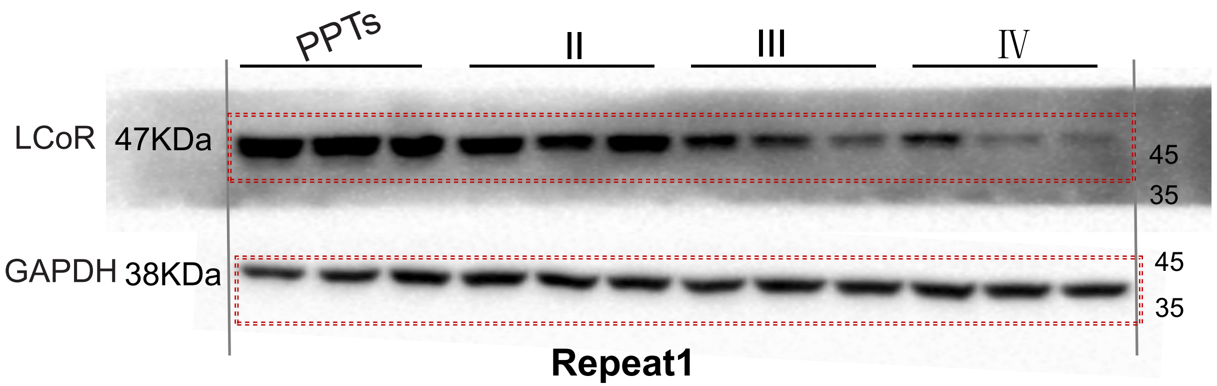

Repeat1

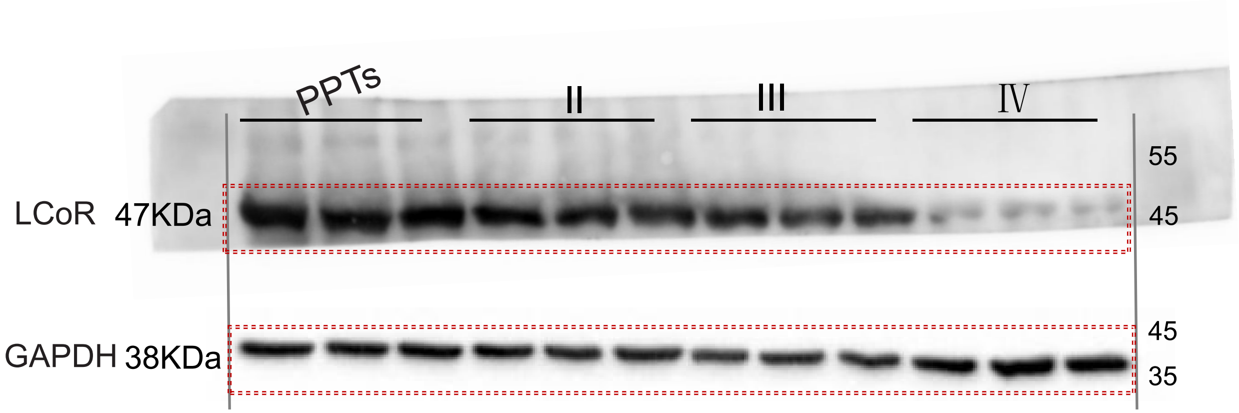

Repeat2

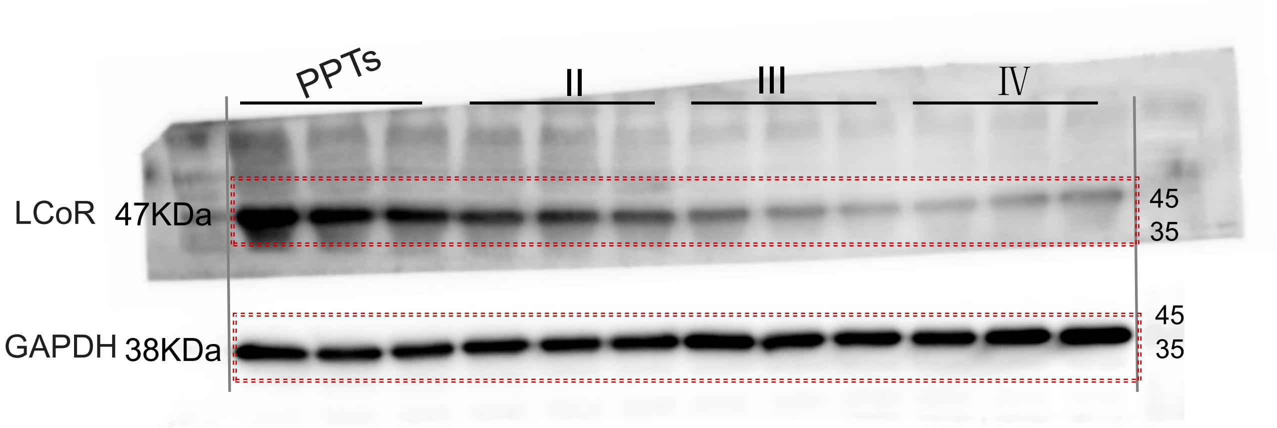

Repeat3

Figure 2A(LCoR)original western blot for three repeats

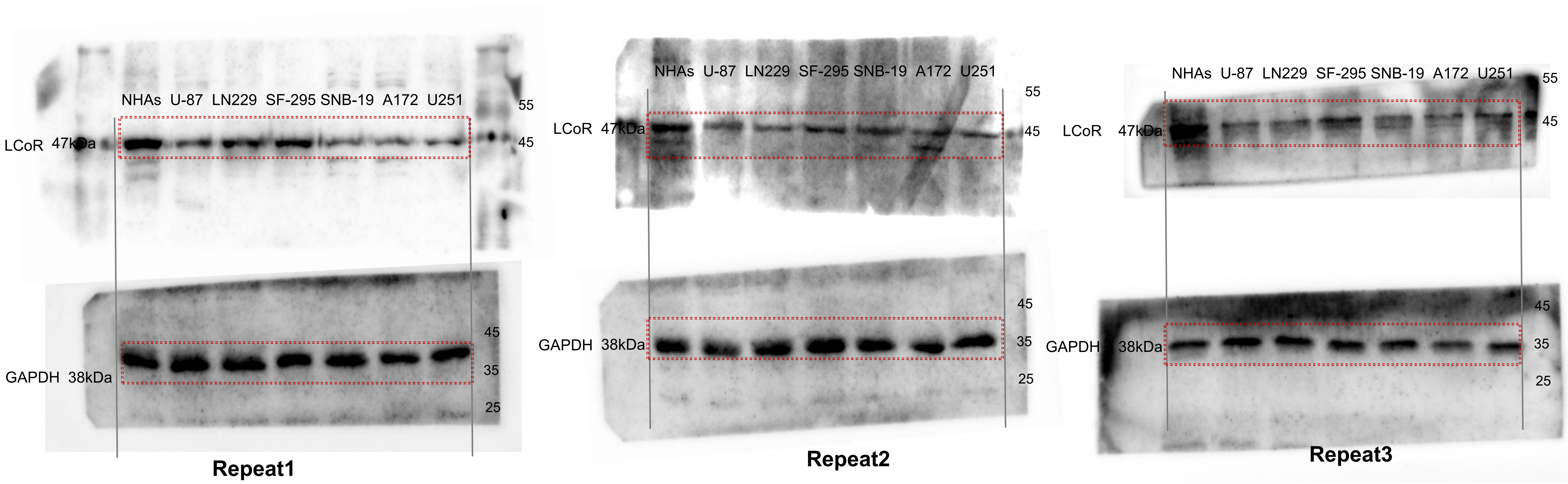

Figure 2B(LCoR)original western blot for three repeats

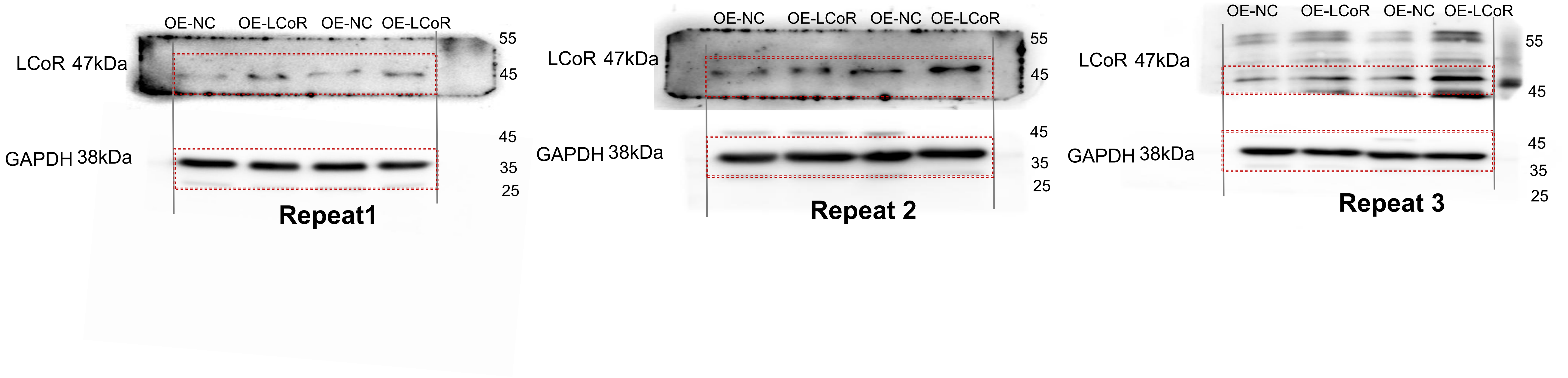

Figure 2C(LCoR)original western blot for three repeats

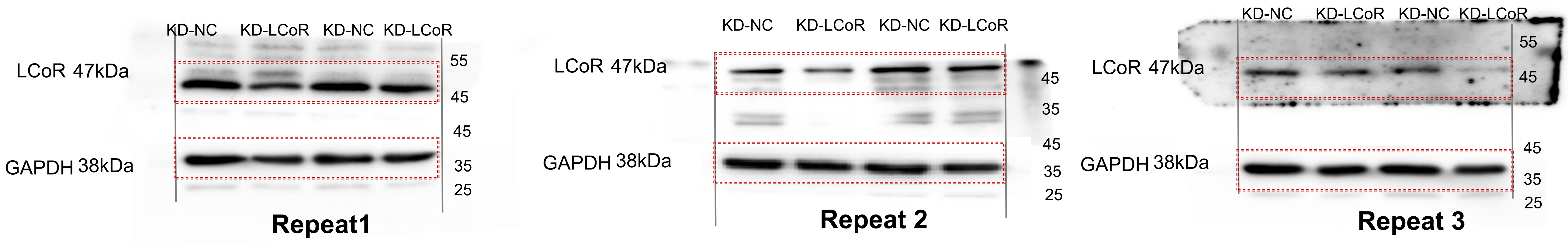

Figure 2H(VEGF/HIF-1α)original western blot for four repeats

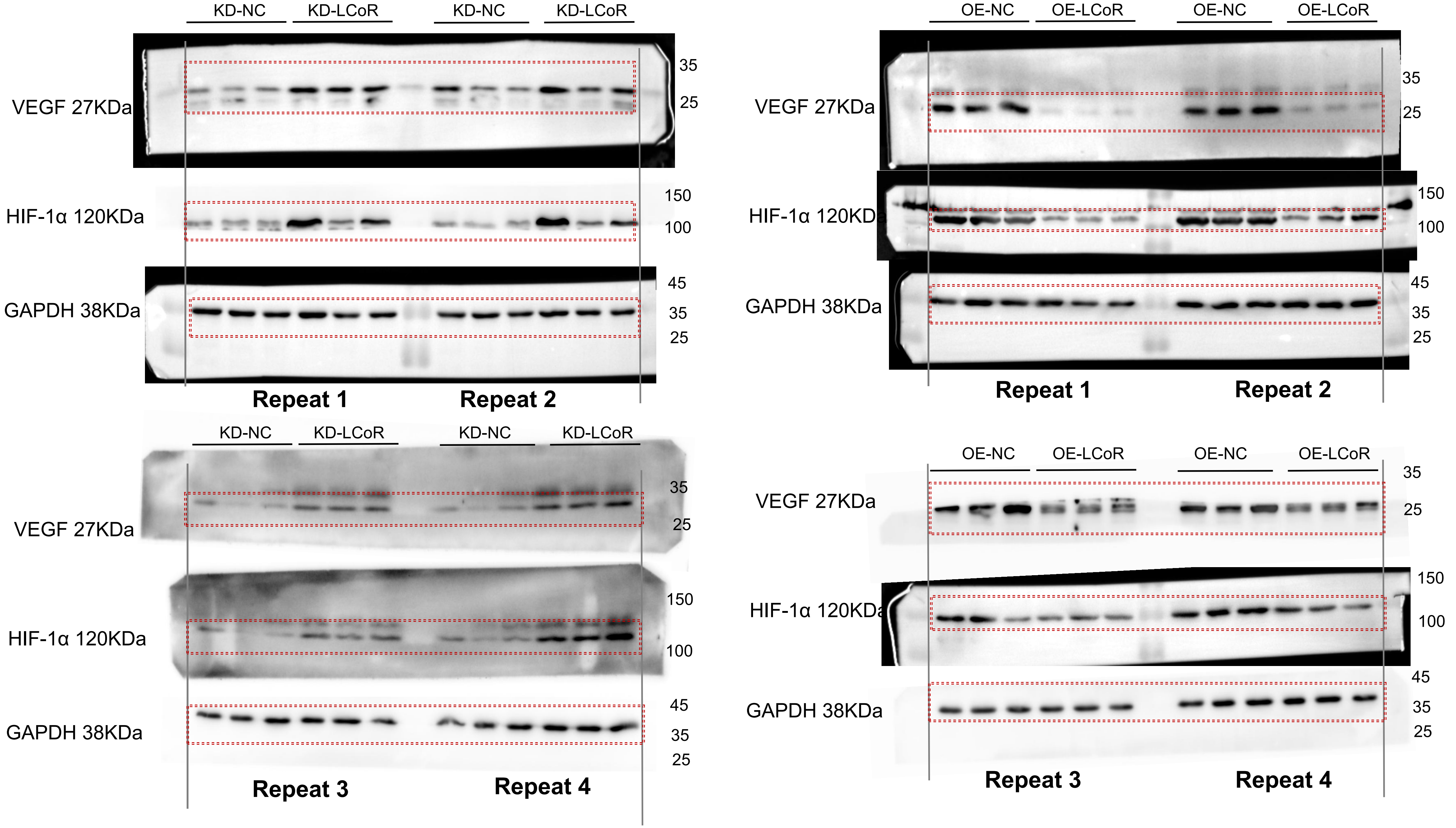

Figure 5F(LCoR/HIF-1α/VEGF)original western blot for three repeats

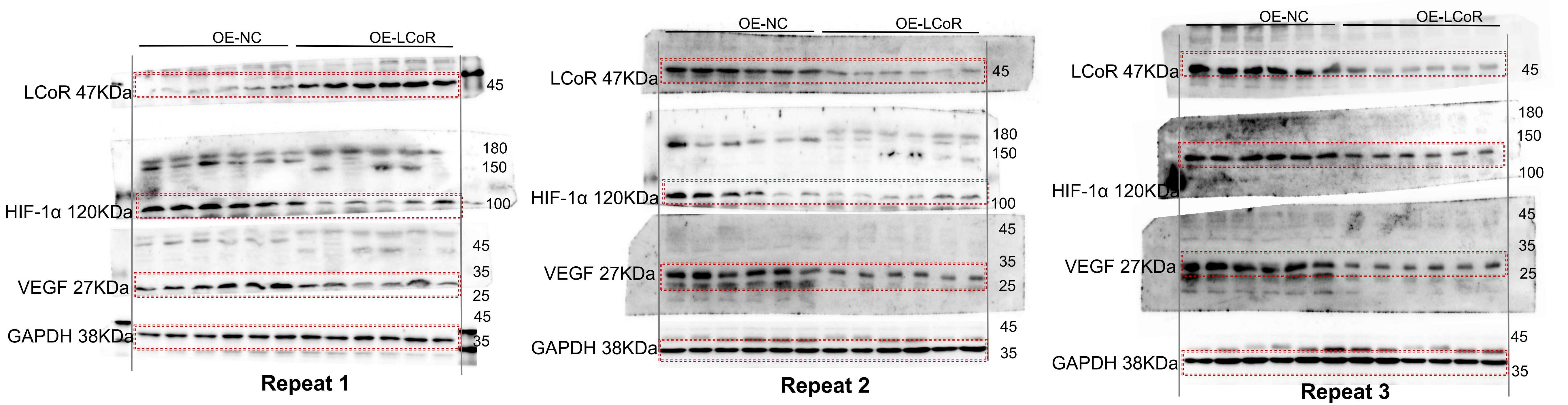

Figure 5K(LCoR/HIF-1α/VEGF)original western blot for three repeats

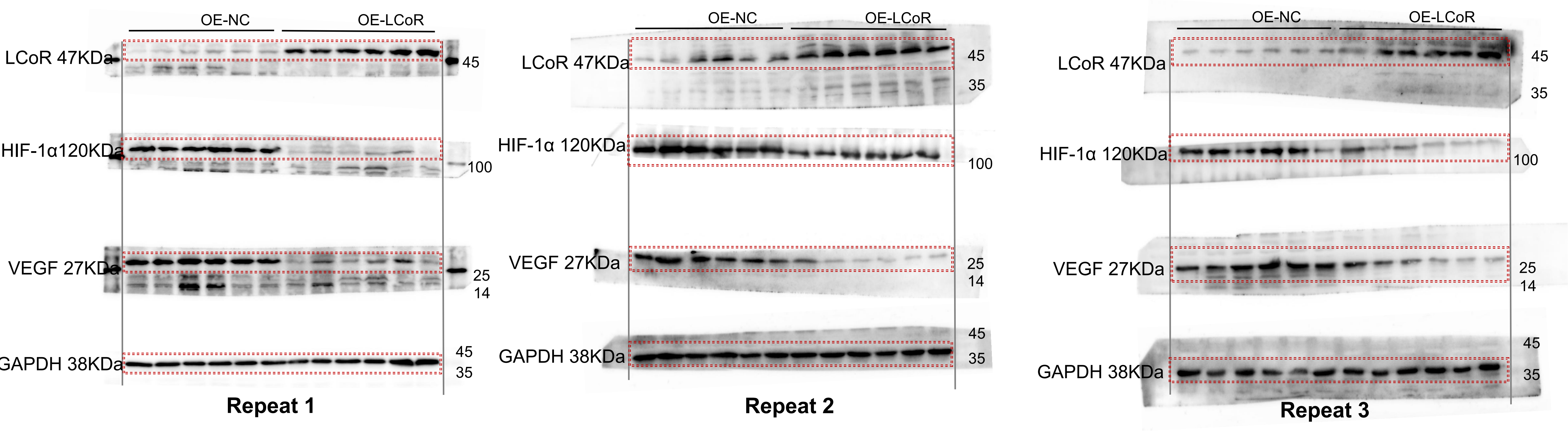

Figure 6E(LCoR)original western blot for three repeats

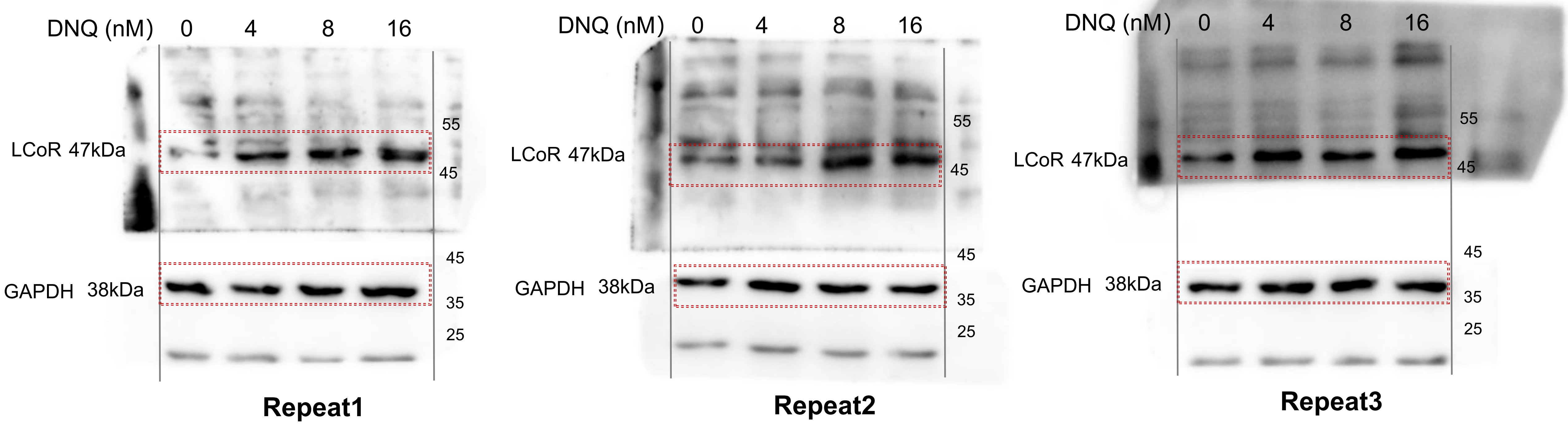

Figure 7H(HIF-1α/VEGF/LCoR)original western blot for three repeats

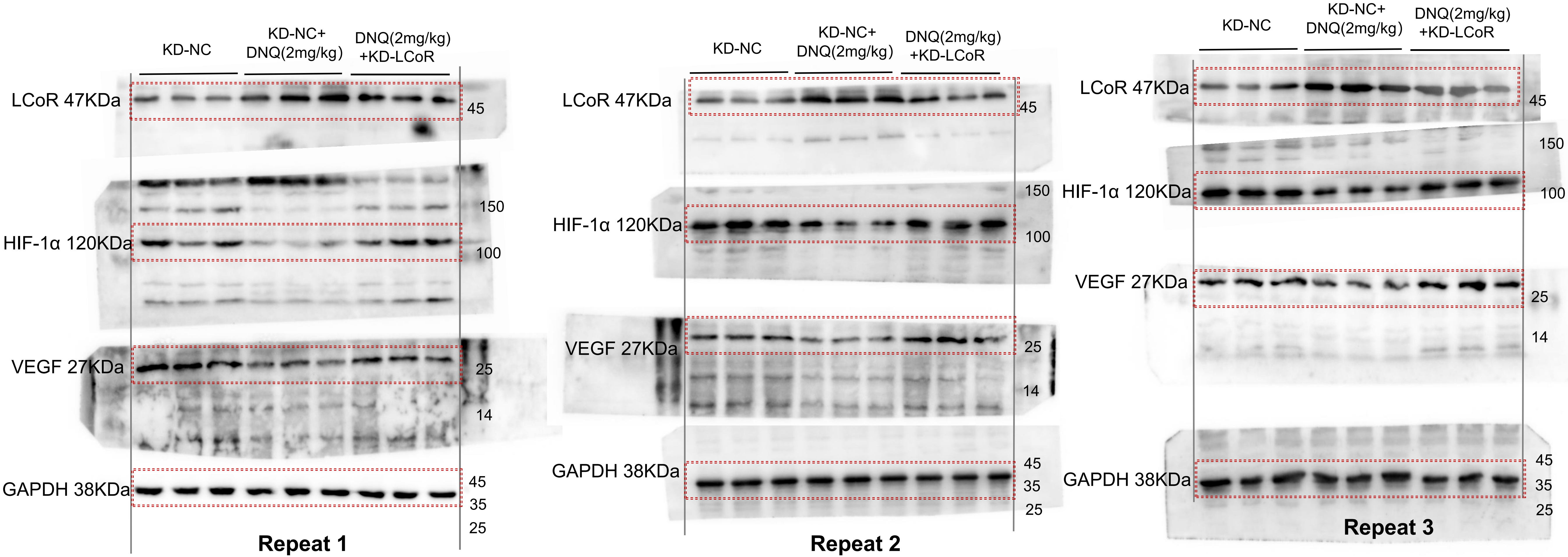

Supplement: Supplementary file 1 — Supplementary Material 1 [file 12896_2026_1166_MOESM1_ESM.pdf]
